# An Electronic Data Capture Framework (ConnEDCt) for Global and Public Health Research: Design and Implementation

**DOI:** 10.2196/18580

**Published:** 2020-08-13

**Authors:** Caleb J Ruth, Samantha Lee Huey, Jesse T Krisher, Amy Fothergill, Bryan M Gannon, Camille Elyse Jones, Elizabeth Centeno-Tablante, Laura S Hackl, Susannah Colt, Julia Leigh Finkelstein, Saurabh Mehta

**Affiliations:** 1 Data Performance LLC Ithaca, NY United States; 2 Division of Nutritional Sciences Cornell University Ithaca, NY United States; 3 Institute for Nutritional Sciences, Global Health, and Technology Cornell University Ithaca, NY United States

**Keywords:** data science, data collection, database management systems, global health, public health, data management, health information management, population surveillance, longitudinal studies, randomized controlled trial, Electronic Data Capture (EDC)

## Abstract

**Background:**

When we were unable to identify an electronic data capture (EDC) package that supported our requirements for clinical research in resource-limited regions, we set out to build our own reusable EDC framework. We needed to capture data when offline, synchronize data on demand, and enforce strict eligibility requirements and complex longitudinal protocols. Based on previous experience, the geographical areas in which we conduct our research often have unreliable, slow internet access that would make web-based EDC platforms impractical. We were unwilling to fall back on paper-based data capture as we wanted other benefits of EDC. Therefore, we decided to build our own reusable software platform. In this paper, we describe our customizable EDC framework and highlight how we have used it in our ongoing surveillance programs, clinic-based cross-sectional studies, and randomized controlled trials (RCTs) in various settings in India and Ecuador.

**Objective:**

This paper describes the creation of a mobile framework to support complex clinical research protocols in a variety of settings including clinical, surveillance, and RCTs.

**Methods:**

We developed ConnEDCt, a mobile EDC framework for iOS devices and personal computers, using Claris FileMaker software for electronic data capture and data storage.

**Results:**

ConnEDCt was tested in the field in our clinical, surveillance, and clinical trial research contexts in India and Ecuador and continuously refined for ease of use and optimization, including specific user roles; simultaneous synchronization across multiple locations; complex randomization schemes and informed consent processes; and collecting diverse types of data (laboratory, growth measurements, sociodemographic, health history, dietary recall and feeding practices, environmental exposures, and biological specimen collection).

**Conclusions:**

ConnEDCt is customizable, with regulatory-compliant security, data synchronization, and other useful features for data collection in a variety of settings and study designs. Furthermore, ConnEDCt is user friendly and lowers the risks for errors in data entry because of real time error checking and protocol enforcement.

## Introduction

### Background

Our research on human nutrition and global health spans multiple countries, encompasses both interventional and observational study designs, and requires secure data collection and management. Noting the benefits of electronic data capture (EDC) over paper-based data capture [1,2], beginning in 2012, we searched for an EDC tool for our research projects on human health and were unable to find a package that met our needs. With several research projects on our roadmap that would be deployed in remote, international regions with complex longitudinal protocols, strict eligibility requirements, and distributed research teams, we aimed to use EDC over paper-based data capture to enforce our study protocols and provide better data security, data validation, and have instant access to data. In surveying EDC packages currently available on the market (Table 1), some had to be ruled out completely because of the unreliable internet coverage in areas we focused on for our studies (compared with Research Electronic Data Capture [REDCap], which had not yet released its mobile app when our initial research projects were being conducted in 2013 [3]). Other packages identified did not provide the required features specific to our study protocols, such as our strict data security specifications (compared with Open Data Kit [ODK]) [4]. To address this, we felt that we had the correct clinical and technical team in place to build our own EDC tools to solve our needs and potentially those of a large community of researchers in the future.

### Objectives

As we developed our plans for creating the EDC tools for our research studies, we decided that instead of a custom tool for each study, a better investment would be one reusable platform that could be applied to each research project, with limited customizations for each iteration. The upfront investment would be higher, but subsequent deployments should build on the initial investment. Mobile phones and tablet devices are commonly used, even in lower-resource settings where our studies are located. We felt that an EDC tool for tablets would be easier for our local research staff to learn, more transportable, and less expensive to deploy than laptops. In this paper, we describe ConnEDCt, the EDC platform that we developed and successfully deployed for clinic-based cross-sectional studies, randomized controlled trials (RCTs), and surveillance projects.

**Table 1:** ConnEDCt, REDCap, and Open Data Kit electronic data capture systems.

Feature	ConnEDCt	REDCap^a^	REDCap Cloud	Open Data Kit 1 Suite, ODK-X^b^ Suite
Requirement to use	FileMaker license	Nonprofit organization with sufficient IT^c^ infrastructure. Join REDCap Consortium, license agreement with Vanderbilt University. Must submit new license to obtain a new REDCap system per each group of users	Fee-based hosting by a third-party company	Web access, user comfort in coding
Mobile app and platform	FileMaker Go on iPhone, iPad	REDCap mobile app; MyCap on iPhone, iPad, Android	REDCap Cloud mobile app on iPhone, iPad, Android	Yes: ODK Connect, Android app
Software and operating system	Claris FileMaker on Mac, Windows	Web server, database server, SMTP^d^ email server, file server (optional) on any laptop	Web server, database server, SMTP email server, fileserver (optional) on any laptop	ODK Aggregate app, ODK Briefcase app, ODK Central app; XML documents created using ODK JavaRosa library/any laptop
Web based	Yes	Yes	Yes	Yes
Offline data capture	Yes	Yes	Yes	Yes
Data synchronization: software	Yes: MirrorSync [5]	Yes	Yes	Yes
Study designs	Customizable	Customizable	Customizable	Customizable
Study protocol enforcement for scheduled and unscheduled encounters	Yes	Not noted	Not noted	Not noted
Electronic-informed consent	Yes	Yes	Yes	Yes
Automated eligibility determination	Yes	Not noted	Not noted	Yes
Randomization	Yes: any form can be implemented	Yes: any form can be implemented	Yes: any form can be implemented	Yes: predetermined types allowed
Conditional CRFs^e^ based on midline serial sampling or other criteria	Yes	Not noted	Not noted	Not noted
Open source	No	No	No	Yes
Regulatory-compliant security and encryption	Title 21 CFR^f^ Part 11, HIPAA^g^, GDPR^h^	HIPAA, Part 11, FISMA^i^ standards (low, medium, or high), GDPR, depending on environment	Best practice security, Title 21 CFR Part 11 compliant, industry regulations, HIPAA compliant, data privacy technology	Security of third-party libraries are not vetted, require user’s security staff to review libraries and source code on GitHub
Secure data collection	Yes	Yes	Yes	Encrypted form security; only transmissions over a secure HTTPS^j^ connection are obscured from observers and prevent tampering in transmission
Export type	Excel, can be customized per user requirement	Excel, PDF, SPSS, SAS, Stata, R	Excel, PDF, SPSS, SAS, Stata, R	Export to CSV^k^, JSON^l^ (text only), KML^m^ (for mapping applications)
Logic checks/concurrent error checking	Yes	Yes	Yes	Yes
Customizable user roles	Yes	Yes	Yes	Yes
Translation and cultural adaptation	Yes	Yes	Yes	Yes
Signature capture	Yes	Yes	Yes	Yes
Ease of form construction	Create using FileMaker software on laptop interface; use Excel to record variable lists; methods adaptable per user’s comfort level	Web-based designer, offline *data dictionary* file on Microsoft Word, Excel	Web-based designer, offline *data dictionary* file on Microsoft Word, Excel	Excel-based form creation (XLSForm), drag-and-drop form creation (ODK Build)
Customizable	Yes	Yes	Yes	Yes
Audit trail	Yes	Yes	Yes	Yes

^a^REDCap: Research Electronic Data Capture.

^b^ODK-X: Open Data Kit-X (formerly known as ODK-2).

^c^IT: information technology.

^d^SMTP: simple mail transfer protocol.

^e^CRF: case report form.

^f^CFR: Code of Federal Regulations.

^g^HIPAA: Health Insurance Portability and Accountability Act.

^h^GDPR: General Data Protection Regulation.

^i^FISMA: Federal Information Security Management Act.

^j^HTTPS: hypertext transfer protocol secure.

^k^CSV: comma-separated value.

^l^JSON: Javascript Object Notation.

^m^KML: keyhole markup language.

## Methods

### Technical Details

ConnEDCt consists of a distributed database, data entry interfaces, data management interfaces, scripted business rules, data synchronization, and data security features [6]. The main components of ConnEDCt are a client-side database, a cloud-based database server, a data synchronization engine, iOS mobile devices, and laptop personal computers. ConnEDCt is built with FileMaker Pro Advanced by Claris International Inc. [7], a commercial, cross-platform, relational database platform. FileMaker Pro Advanced provided the important advantages of rapid development and the ability to deploy cross-platform on iOS, Mac, and Windows platforms. One tool is therefore easily deployed on iPads and laptops, which are our primary computing devices. ConnEDCt’s features, quality, and safety are reliable compared with existing guidelines and requirements, including Good Clinical Data Management Practices [8], the Food and Drug Administration (FDA) [9], and Title 21 Code of Federal Regulations (CFR) Part 21 [10].

### Configuration of ConnEDCt

Study designers conduct a design process using Microsoft Excel templates to define the study schema. A *forms schedule* template is used to define case report form (CRF) usage over scheduled visits in a longitudinal study. The intersection of CRFs and scheduled visits in a two-dimensional matrix in this template shows which CRFs are instantiated per visit. In addition, CRFs can be defined for unscheduled instantiation. Study designers use *data dictionary* Excel templates to define variables within CRFs along with select lists, skip logic, etc. The study designers then document the eligibility criteria formulas within another Excel template.

The data dictionaries are then translated into FileMaker tables and fields in a FileMaker template file. FileMaker provides an easy-to-use interface for creating CRF tables and fields within the integrated development environment. A study designer can accomplish this step with minimal training. The final steps of implementation require some developer skills and are easily repeatable within a framework structure. These implementations include the forms schedule schema, eligibility criteria formulas, CRF validation, loading a randomization table, and synchronization. Once ConnEDCt has been configured for a study, the server files are then hosted on an internet-connected server, and client files are deployed to laptops and iPads.

We continue to develop the features of ConnEDCt further to enable study designers to complete the implementation of the majority of study protocols with minimal developer involvement. We anticipate that to maximize the flexibility of study protocols, we will always benefit from some custom or specialized development for novel EDC features.

### Key Features

#### Management of Research Teams and Different Roles

Several *roles* were required to implement and operate ConnEDCt. *Software developers* and *systems integrators* performed programming and other technical tasks such as database and server management. *Data managers* helped translate clinical requirements into data definitions and used software interfaces to define the scheduled and unscheduled study events, CRFs, variables, validation criteria, and eligibility criteria. Each study event involves capturing data in one or more CRFs. A CRF is a collection of related variables to be entered during a study event. The data manager also manages data exports for analysis by using external statistical and analytics software. *Research associates/assistants* perform data entry during interviews, direct observations, and also enter laboratory results. User-level privileges to various database features and data vary by role. For example, *research assistants* may create new participants and enter data, whereas research coordinators and principal investigators may additionally edit the entered data in the event of errors found as data managers. Different users can access ConnEDCt with their own account, secured by a custom username and password, which contains different levels of privileges depending on the user role.

#### Asynchronous Data Synchronization

A key feature of ConnEDCt is the ability to capture data on a mobile device while offline and, at a later time, when an internet connection is available, send the captured data to a cloud-based server. In addition, study schema revisions can be deployed on mobile devices. The asynchronous data transport can be finely controlled such that data can be selectively deployed to individual mobile devices. In this manner, data can be synchronized in 1 direction only to minimize data traffic and to maintain data privacy. Data previously stored in mobile devices will remain in the FileMaker Go app on mobile devices to enable research assistants to track participant progress throughout the study. After study completion, the data are completely removed from mobile devices.

#### Real-Time Assessment of Eligibility

**Figure 1:**
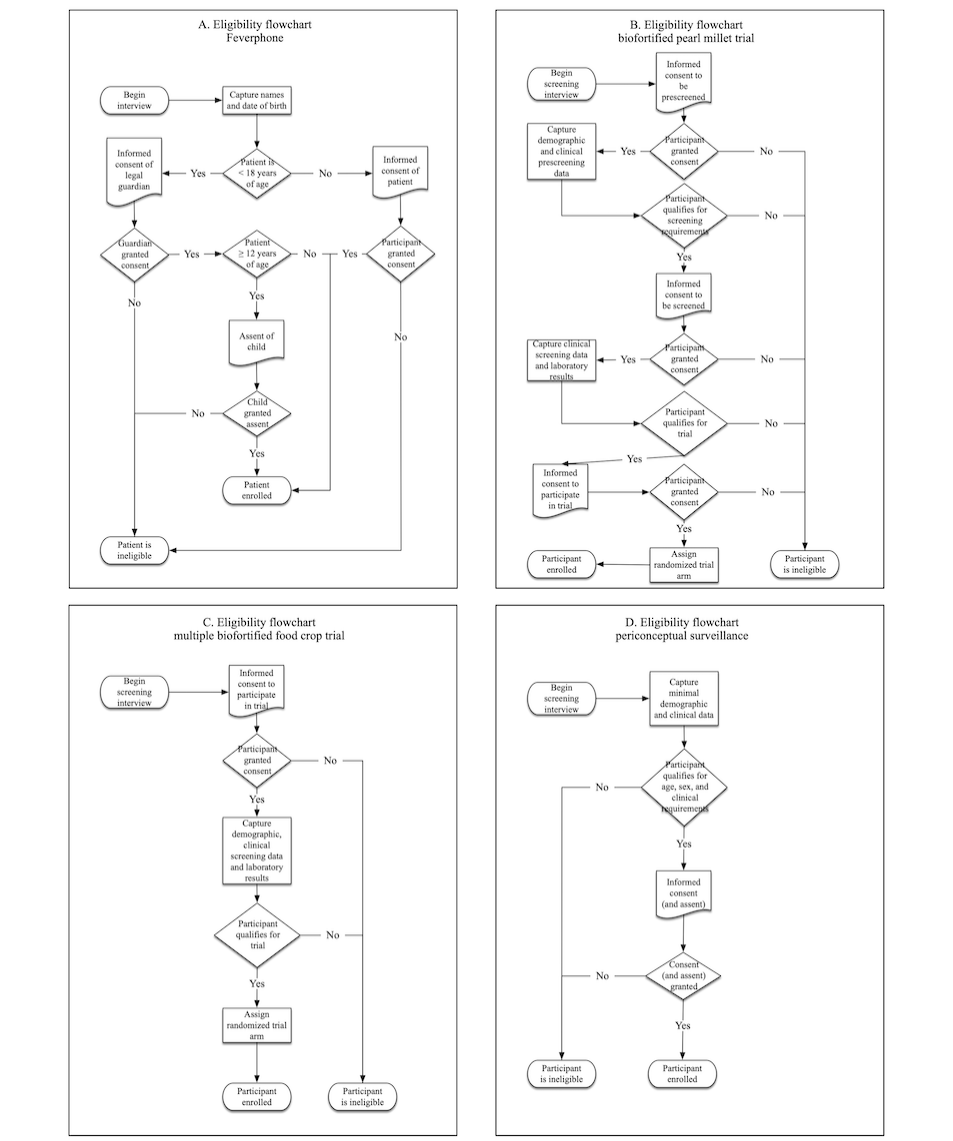
Eligibility protocol for (A) clinical setting: FeverPhone, Ecuador; (B) RCT, Mumbai; (C) RCT, South India; (D) surveillance study, South India. RCT: randomized controlled trial.

Assessing eligibility is a key process in participant screening and study enrollment. ConnEDCt provides automated features to determine participant eligibility based on criteria specified a priori. Eligibility algorithms are evaluated programmatically at runtime to provide feedback on participant eligibility. Figure 1 shows the workflow for evaluating the eligibility of a new participant. As eligibility criteria may be defined by including variables from different CRFs, eligibility is evaluated on completion of each predicate CRF. Participant exclusion can be determined based on a negative evaluation of a single criterion, whereas inclusion is determined on completion of all predicate CRFs.

#### Electronic Consent and Signatures

Electronic signatures and health records are regulated under the *FDA Title 21 CFR Part 11 of the CFR* *of the United States* [11]. *Part 11* requires strict data security, including user authentication, encryption, and auditability. Data security regulations vary by country, and ConnEDCt is compliant with currently known rules and adaptable to potential future data security requirements. For example, when study protocols require audiovisual evidence of consent, ConnEDCt can use a mobile device’s built-in camera and microphone to capture the consent process.

#### Enforcement of Complex Study Protocols

**Figure 2:**
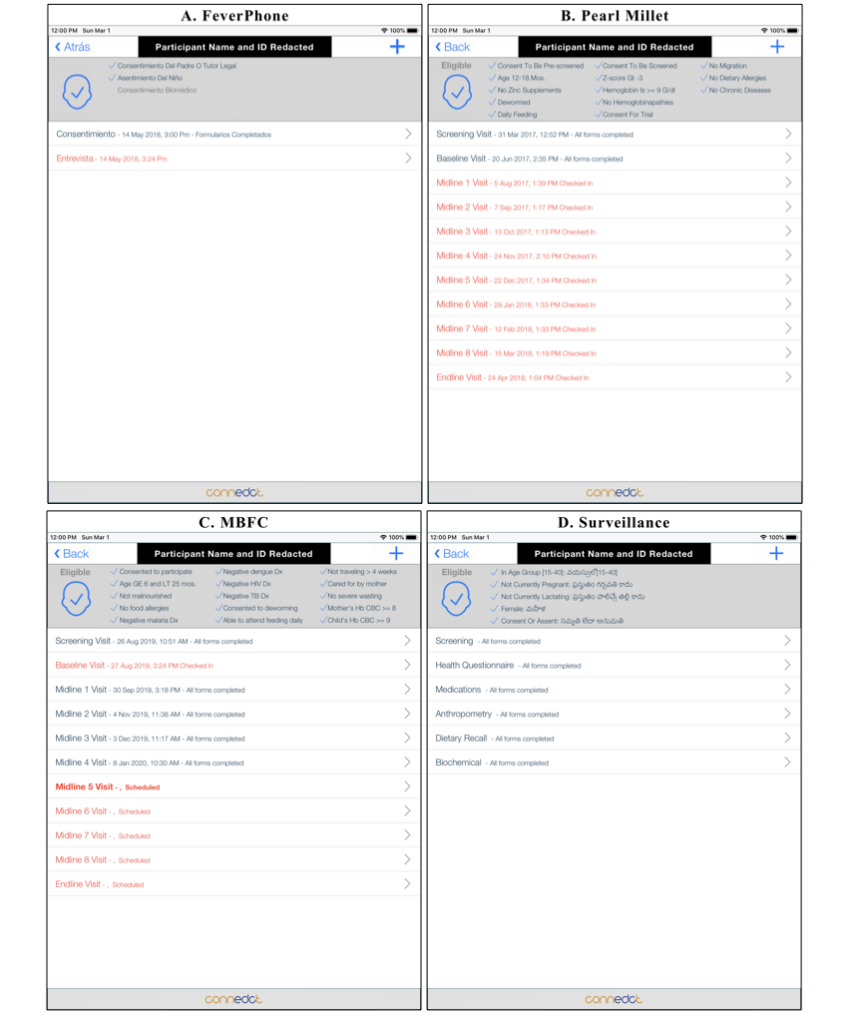
Schedule of visits for (A) clinical setting: FeverPhone, Ecuador; (B) RCT, Mumbai; (C) RCT, South India; (D) surveillance, South India. RCT: randomized controlled trial.

**Figure 3:**
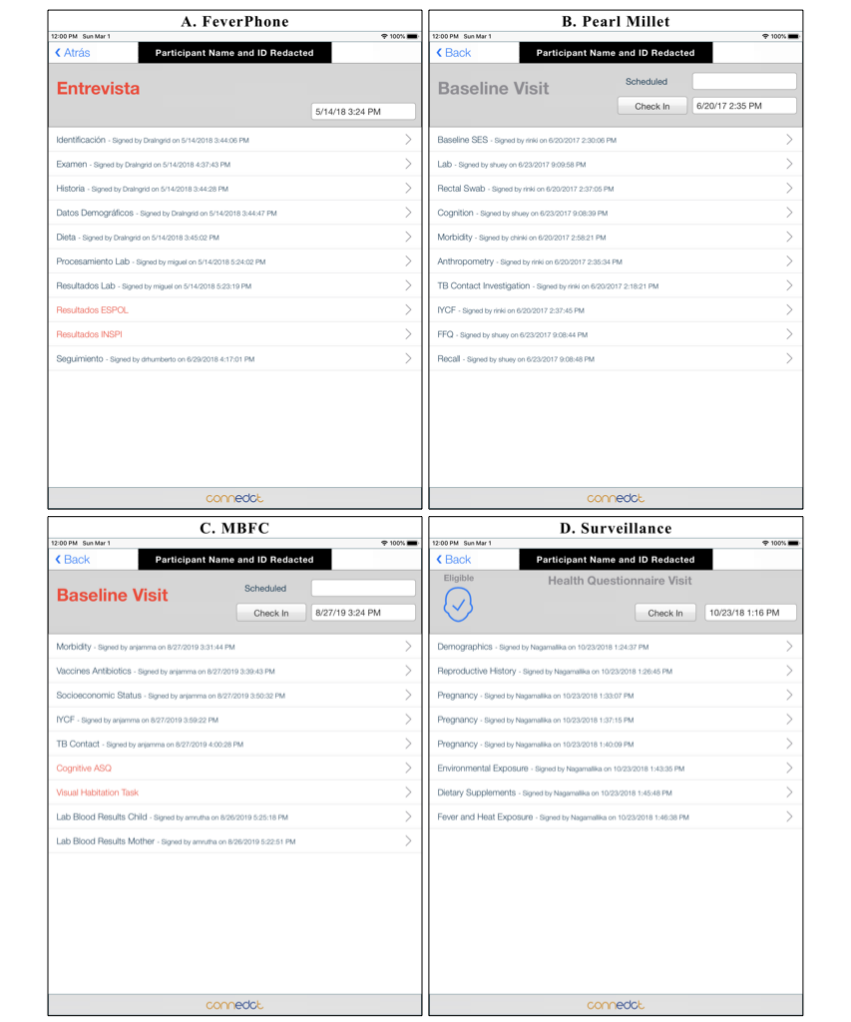
Schedule of visits for (A) clinical setting: FeverPhone, Ecuador; (B) RCT, Mumbai; (C) RCT, South India; (D) surveillance study, South India. RCT: randomized controlled trial.

**Figure 4:**
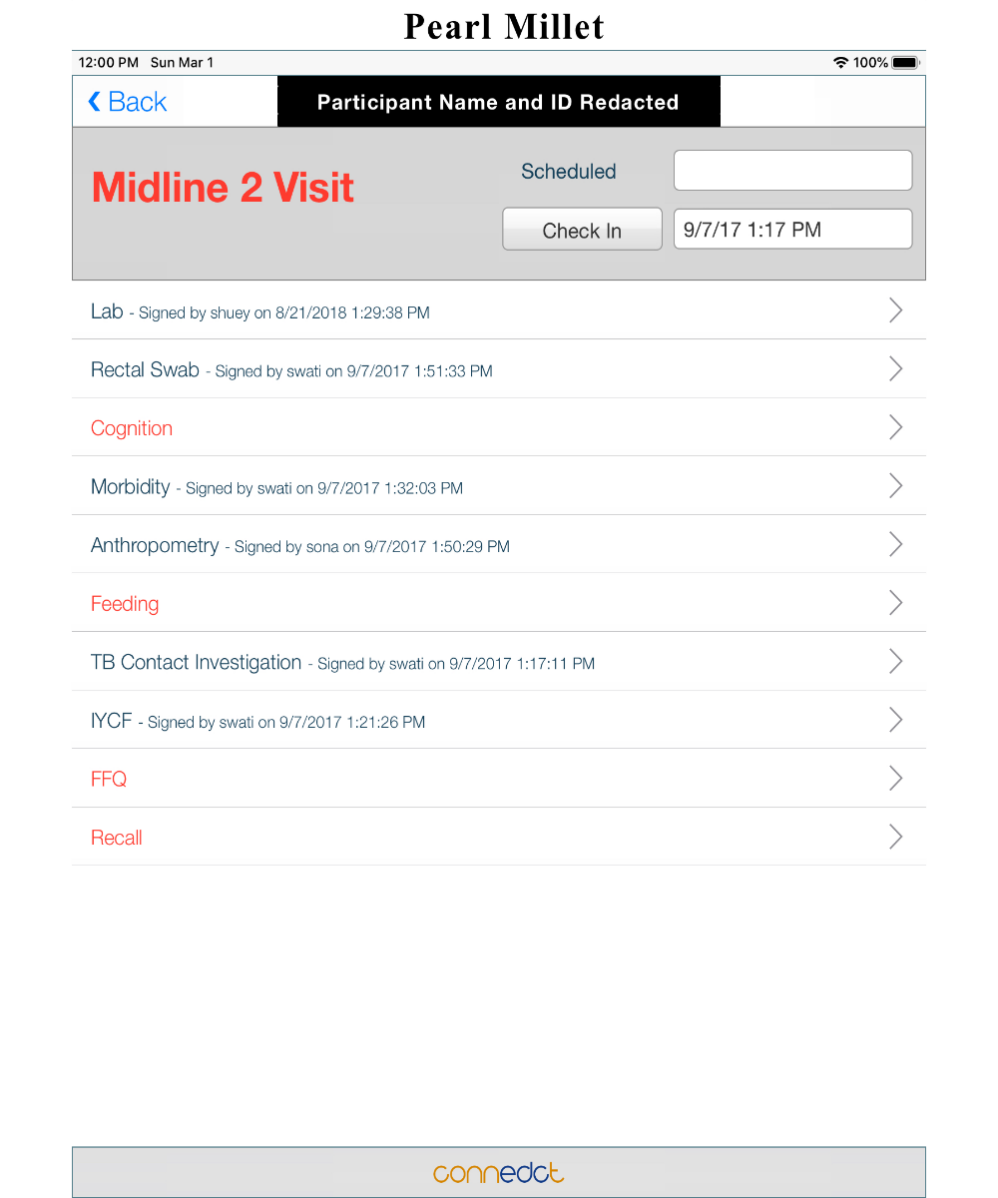
Example of display of all CRFs required for (A) baseline visit, for clinical setting: FeverPhone, Ecuador; (B) baseline visit for RCT, South India; and (C) health questionnaire visit for surveillance, South India. CRF: case report form; RCT: randomized controlled trial.

We designed ConnEDCt to support complex, longitudinal study protocols and minimize training requirements for field research staff. ConnEDCt provides features to support predefined longitudinal study schema, automated assignment of randomized study arms and midline serial sampling, and automated scheduling of additional CRFs based on captured responses. When ConnEDCt is configured for a study, visit records (eg, screening, baseline, midline, and endline) will be created as dictated by the study schema. CRFs are organized within appropriate visits. A visit is complete when all CRFs within the visit have been completed and signed. The interface clearly shows the schedule of visits (Figure 2) and the required CRFs for a particular visit (Figure 3). When the study includes an RCT, ConnEDCt can reference a randomization table to automatically assign a randomized arm to an eligible participant. When the study protocol includes midline serial sampling, ConnEDCt uses the internal randomization table to selectively include designated CRFs at the randomly designated midline visit (Figure 4). When responses to study questions determine the need for additional CRFs, they will be created on demand and presented in the visit schedule—for example, a response to a question on the number of pregnancies may trigger the same number of pregnancy CRFs, or a response on a sick form may trigger a blood or saliva sample CRF. These features reduce the learning curve for the research team. We believe that this improves the interview experience by allowing research assistants to focus on the study participant instead of on data management and study protocol compliance.

#### Designed for Flexibility and Reuse

The ConnEDCt architecture has a flexible schema and is designed to be adaptable to varying study protocols. Therefore, ConnEDCt is designed for flexibility and reuse. Events, event types, event forms, form schedules, form types, and eligibility criteria are defined in the data model and can be modified in the user interface. Validation rules and postprocessing triggers are built in a framework, minimizing the amount of custom code. In this manner, subsequent research studies can be implemented with minimal modification to the conceptual schema or programmatic code. When a new study protocol is implemented, entities for visits and CRFs are created, whereas the programming that controls participant management, navigation, eligibility, and study protocols responds to changes in study protocol design.

#### Language Customization and Data Location

**Figure 5:**
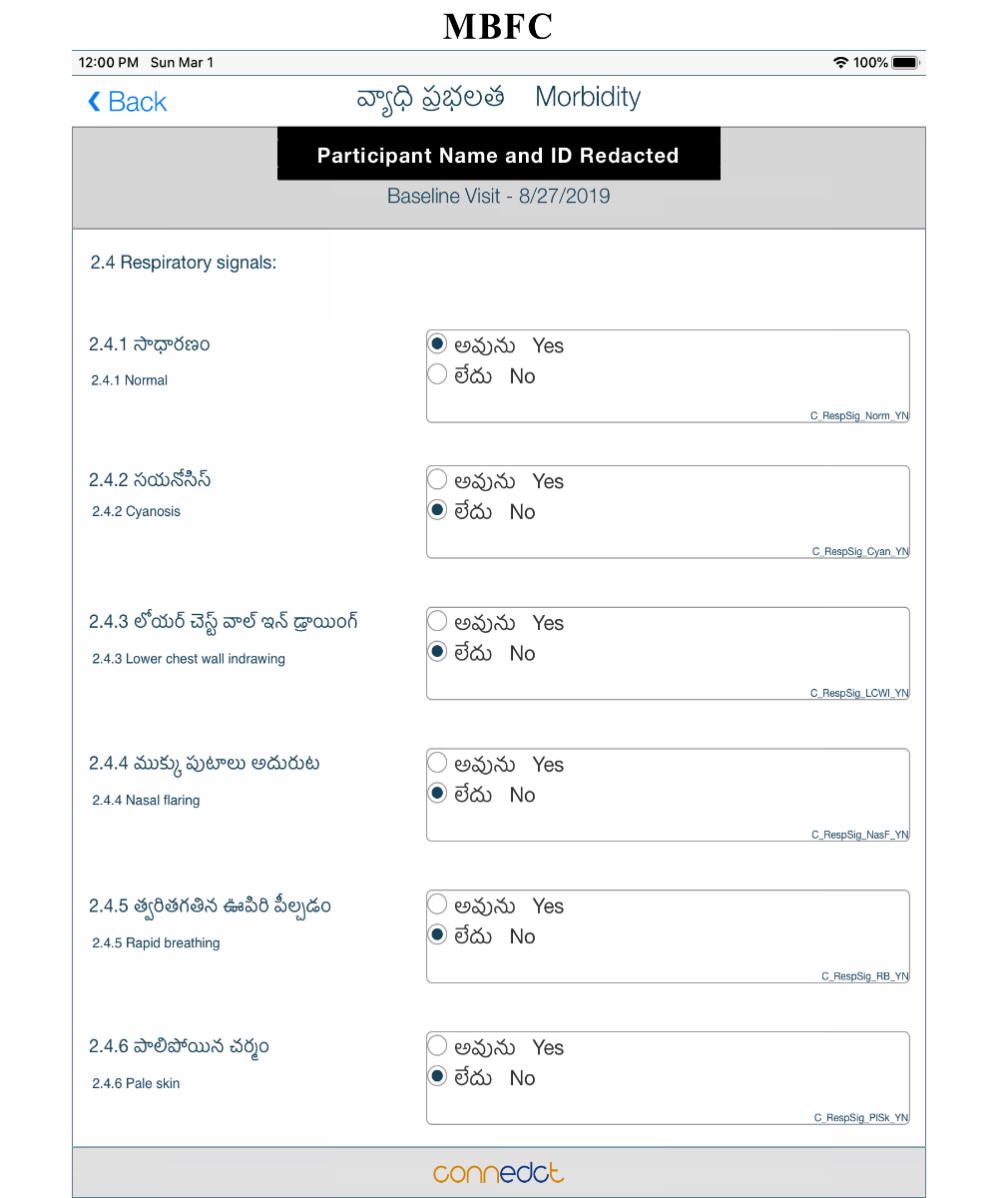
CRF shown in the local language, Telugu, for RCT, South India. CRF: case report form; RCT: randomized controlled trial.

The ConnEDCt system can also accommodate CRFs in multiple languages, as shown in Figure 5. Professionally translated questionnaires can be entered into FileMaker using native multilingual keyboards. Furthermore, data can be synchronized to and primarily stored on in-country servers, where available, to comply with local laws in many settings.

## Results

### Description of Current Implementation: Case Studies

The ConnEDCt platform has been used in a variety of contexts within our research teams, including cross-sectional studies in clinical settings, surveillance studies (a repeated cross-sectional study in the clinic or the community, the latter surveying thousands of households), and RCTs (Table 2).

**Table 2:** ConnEDCt usage in case studies: clinic-based cross-sectional studies, surveillance studies, and randomized controlled trials.

Study design	Clinic-based cross-sectional study	Surveillance (repeated cross-sectional studies)	RCT^a^	RCT	RCT
Location	Ecuador	South India	Mumbai	South India	South India
Study reference	FeverPhone	Periconceptional program	Biofortified pearl millet	Multiple biofortified food crops	Quadruple fortified salts
ClinicalTrials.gov ID	N/A^b^	NCT04048330	NCT02233764	NCT02648893	NCT03853304
Status	Active	Active	Complete	Active	Planned
Synchronization methods	Continuously, using mobile SIM card, simultaneously from multiple locations	Daily, after data collection	Daily/weekly, after data collection	Daily, after data collection	Daily, after data collection
Number of participants entered into ConnEDCt	Overall 404 children and adults (including pregnant women)	2404 households (2876 women)	407 children	345 mother-infant dyads	1000 women of reproductive age
Informed consent process	Separate consent or assent forms depending on participant’s age	Complex, depending on participant’s age (estimated or actual)	3 forms (prescreening, screening, enrollment)	1 form, with multiple levels of consent	Complex, depending on participant’s age (estimated or actual)
Eligibility	Evaluated in real time on completion of CRFs^c^	Evaluated in real time on completion of CRFs	Evaluated in real time on completion of CRFs	Evaluated in real time on completion of CRFs	Evaluated in real time on completion of CRFs
Unique features	Continuous synchronization across multiple locations simultaneously; ability for forms to be signed by multiple study personnel to trace missing information	Only requisite CRFs appear for research staff to complete; complex algorithm for study ID creation	Midline serial sample randomization scheme	2-level randomization to midline serial sample	Only requisite CRFs appear for research staff to complete; randomization scheme to establish 2×2 factorial design
Data types entered	Laboratory assay results, growth measurements, sociodemographic data, health history and current clinical signs, dietary frequency, and biological specimen collection dates and processing	Laboratory assay results; anthropometric measurements; sociodemographic data; complete reproductive history; general health history and clinical signs and symptoms; 24-hour recall; risk factors for birth defects (environmental exposures, medication use, and history of fever); and biological specimen collection volume, dates, and storage conditions	Laboratory assay results; growth measurements, sociodemographic data; infant and young child feeding; health history and clinical signs and symptoms; biological and specimen collection dates	Laboratory assay results; growth measurements, sociodemographic data; infant and young child feeding; health history and clinical signs and symptoms; and biological specimen collection dates	Laboratory assay results; anthropometric measurements; sociodemographic data; complete reproductive history; general health history and clinical signs and symptoms; 24-hour dietary recall; food frequency questionnaire; risk factors for birth defects (environmental exposures, medication use, and history of fever); and biological specimen collection volume, dates, and storage conditions
Language customization	Spanish	Telugu	Hindi	Telugu	Telugu

^a^RCT: randomized controlled trial.

^b^N/A: not applicable.

^c^CRF: case report form.

### Clinical Research: Clinic-Based Cross-Sectional Studies

ConnEDCt has been the primary data collection tool for clinic-based cross-sectional studies in clinical settings of Ecuador for the National Institutes of Health-funded project *FeverPhone: Point of Care Diagnosis of Acute Febrile Illness using a Mobile Device* and was integral in the development of the Ecuador FeverPhone project protocols and manual of operations. Multiple types of users, based at different locations, input data into separate tablet computers with distinct CRFs on participant recruitment, eligibility, and informed consent; multiple participant study visits; and specimen management and laboratory results. The integration of ConnEDCt across devices with daily synchronization of data to a cloud database and local computers allows research staff to easily review data for quality control, implement protocol revisions, and share progress with international collaborators.

### Surveillance Research: Repeated Cross-Sectional Studies in a Clinic or the Community

The ConnEDCt platform provides the foundation for a periconceptional surveillance program, *Periconceptional Surveillance for Prevention of Anemia and Birth Defects in India,* funded by the US Centers for Disease Control and Prevention. This preintervention biomarker survey is being conducted among 1500 women of reproductive age (15-40 years) who are not pregnant or lactating along with their households in Southern India (ClinicalTrials.gov ID: NCT04048330; as reported in Finkelstein JL, Fothergill A, Johnson CB, et al., 2020). ConnEDCt allows enumerators to select from an imported list of households and to individually screen all women from each household while integrating all household-level data into each woman’s final record. Synchronization is performed daily after data collection on all iPads. This allows for the maintenance of all household characteristics for women in the study while preventing enumerators from needing to re-enter data for all women within a household.

### RCTs: Intervention Studies

The ConnEDCt platform for surveillance described above will be incorporated for an upcoming randomized efficacy trial, *A Randomized Trial of Quadruple Fortified Salt for Anemia and Birth Defects Prevention in Southern India* (ClinicalTrials.gov ID: NCT03853304; quadruple fortified salt [QFS]). This trial will be conducted among 1000 women of reproductive age who are not pregnant or lactating with their households using similar algorithms for eligibility and CRFs for data collection as the surveillance study.

ConnEDCt was also used for an RCT in Mumbai, *Effect of Iron/Zinc-biofortified Pearl Millet on Growth and Immunity in Children Aged 12–18 Months in India* (ClinicalTrials.gov ID: NCT02233764) that completed data collection in July 2018 [12]. The full protocol of the study was integrated into ConnEDCt, which included the eligibility evaluation of over 400 participants; randomization to one of 2 experimental arms, accounting for a random midline serial sample; and a diverse set of CRFs to collect data from participants at 9 monthly follow-up visits. After a training session, local research assistants independently scheduled visits and collected data throughout the trial, including synchronization to the secure server. Importantly, ConnEDCt was also able to be modified after pilot testing among research assistants and participants in the field to improve the workflow, such as improving the placement of certain buttons or options on each form screen. Real-time feedback of data throughout the trial allowed for error checking concurrently with data collection. Specifically, data were exported from ConnEDCt as a relational database in the form of an Excel spreadsheet (1 per each type of CRF) using a custom script. These data were then imported and merged into 1 full database in statistical software packages and subsequently analyzed for errors, such as biologically implausible values or typographical errors during data entry. One asset of the ability to analyze the data in SAS concurrently with the trial permitted examination of the raw variables for a particular eligibility criterion necessitating advanced statistical analysis, after which results could be re-entered into ConnEDCt. The ability to access the full database concurrently as the trial was ongoing was also crucial to summarizing data, such as technical reports to the study funders and progress reports, as well as any adverse events for our data safety and monitoring board.

The EDC framework established for the Mumbai trial was customized and expanded for a second ongoing RCT evaluating multiple biofortified food crops (MBFC) in children and their mothers, *Effect of a Biofortified Food Basket on Micronutrient Status and Immune and Cognitive Function among Infants in India* in Madanapalle, South India (ClinicalTrials.gov ID: NCT02648893) to incorporate a different type of participant (mother-child dyads) and CRFs specific to the trial. The protocol was modified to include (1) a 2-level randomization to 1 of 4 color-coded groups, with 2 groups corresponding to each treatment arm and randomization to a midline sampling point; (2) visit scheduling and tracking over a 9-month follow-up period; and (3) data collection across integrated CRFs to handle screening and eligibility determination, sample collection, and participant tracking. A randomization protocol was created that was executed by the study statistician and shared with the ConnEDCt programmer to incorporate into the database. Similar to Mumbai, the MBFC trial is synchronizing daily after data collection during screening, baseline, and follow-up appointments. 

### Errors During Implementation

During implementation of data capture, the more common errors included inputting the *wrong* CRF for a participant at the wrong visit or miscoding variables (eg, a numeric coding for a character variable). These were easily resolved by the database developer and/or data manager.

## Discussion

### Principal Findings

The benefits of using ConnEDCt as an EDC system greatly outweigh the few challenges. Below, we describe how we addressed potential or actual issues of connectivity, complexity in consent forms, participant confidentiality, and research staff compliance.

### Problem 1: Connectivity

In all settings, data were synchronized to the server and the participant’s data were harmonized by the MirrorSync software [5]. To account for lapses in internet connectivity and prevent data conflicts, synchronization was performed at the end of each day of data collection in the RCTs to avoid data conflicts and any need for internet connectivity throughout the data collection process. In practice, 1 iPad was assigned to a particular participant for the duration of the visit and, after completion of the visit, all iPads were synced to the data server.

In contrast to RCTs, the clinic-based cross-sectional study in Ecuador (FeverPhone) required multiple users to input data for a single participant using separate devices in different clinical locations. Therefore, regular synchronization was important for study procedures. The clinical site did not offer wireless internet access, but each iPad was fitted with a prepaid mobile SIM card to allow for regular synchronization during participant visits.

For the periconceptional surveillance program, the database was designed such that iPads could remain at 1 station for data collection while participants moved from station to station. This greatly simplified data collection and allowed each enumerator to continue using the same iPad to help track enumerator productivity and workflow.

### Problem 2: Complex Consent Forms

In Mumbai’s RCT, informed consent was taken at 3 separate visits: prescreening (consent to noninvasive data collection), screening (consent to biospecimen collection and growth measurements), and enrollment (consent to participate in the trial), including informed consent signatures per the type of data collected. During follow-up, we included informed consent signatures on biological specimen collection forms to remind participants of their option to opt out of the trial at any time.

One RCT in Southern India, the MBFC trial, combined the informed consent process into a single form, with multiple levels of consent. Participants were asked separately for their consent to screening and participation in the trial, subsequent biological sample storage and analysis, and future analyses of their biological samples, which may include genetic analysis. This allowed documentation and tracking of different levels of consent for each participant as well as allowing for tracking of which participants consented to future analyses.

At the Ecuador clinical site, study procedures required separate consent or assent forms depending on the participant’s age. ConnEDCt automatically provided the appropriate consent or assent forms based on the date of birth information provided in the recruitment CRF. ConnEDCt would further limit progress to subsequent CRFs if the appropriate consent or assent forms were incomplete.

The periconceptional surveillance study in Southern India has a complex informed consent process because of multiple factors influencing eligibility and additional factors to determine which set of informed consent forms need to be completed. In the periconceptional surveillance program, participants included adolescents aged 15 to 18 years (requiring an assent form and parental consent) and adults (≥18 years of age, requiring a consent form) are potentially eligible to participate. To ensure that participants complete the correct forms and that all required signatures are obtained, the participant’s age (to the day) is required in real time. ConnEDCt was able to be adapted for this purpose to determine a participant’s eligibility, and which set of forms they needed to complete to enroll. Additionally, to account for variable amounts of information being available for the determination of current age, we developed an algorithm that considered all available information (current age, exact date of birth, and estimated/partial date of birth). The database developer built the algorithm into ConnEDCt such that research assistants entered data available to them, and the software made visible the CRFs that they needed to proceed (eg, CRFs indicating the participant was eligible and which set of assent/consent forms they needed to sign). To simplify and streamline the process, we adapted ConnEDCt such that it only allowed necessary CRFs to appear on the iPad for the research assistants, making it impossible for research assistants to fill out the incorrect set of CRFs.

### Problem 3: Confidentiality

Confidentiality was maintained in RCTs, clinic-based cross-sectional studies, and repeated cross-sectional studies, such as our surveillance program, through many layers of security. First, each field personnel had their own computer-generated unique username and password to access the database. Second, although participant names were included in the database to facilitate tracking of each participant locally, names were easily removed for deidentification after exporting the data. Participants were identified with ID numbers derived from a 4-digit sequentially generated code and a letter corresponding to the participant’s color group study allocation.

The periconceptional surveillance program includes women of reproductive age and their families—and a hierarchical data structure. In the database, each participant has an identifying individual participant ID and a household ID, which in combination, uniquely identifies them as a participant in the periconceptional surveillance program; the latter being generated by ConnEDCt. Their personal ID incorporates a code that indicates that they are a participant in the surveillance program, a code indicating which iPad their data were collected on, and an additional 4-digit code (generated sequentially by ConnEDCt) to ensure that all participants have unique IDs. This identification system in ConnEDCt allows for an undetermined number of participants to be enrolled in the surveillance program while ensuring that no duplicate IDs are utilized. The structure of the IDs (3 concatenated codes) also provides flexibility to add and remove pieces to indicate the person’s participation in potential future studies led by the Finkelstein research group in this area (eg, for the upcoming QFS randomized efficacy trial).

### Problem 4: Compliance

The data collected in the periconceptional surveillance program are very detailed and complex. To improve data quality during collection, we built various safeguards into the data collection tools. For free-entry numeric variables, limits were placed around values entered that would flag responses and indicate enumerators that data entered seemed implausible. For example, if a participant’s weight was entered as 1 kg, a flag would pop up on the iPad screen, indicating that the response was outside the anticipated range and ask the enumerator to verify the entry before moving forward. In addition to raw variable entry, these flags were also built in to read across multiple variables that were deemed to be of high importance. If a participant’s response was left blank, or if a response to question 1 directly contradicted with an entry for question 3, a flag would pop up and ask the enumerator to verify before continuing. Finally, various time stamps were built into variables throughout the questionnaire to allow for remote monitoring of enumerator progress. Enumerators had to log in using their unique username and password before any data entry and had to sign each form they entered data on using their unique username and password before they could continue.

The RCTs incorporated similar compliance measures into the ConnEDCt EDC system, including real-time calculations for replicate measurements, thresholds for variation to prompt additional replicates, and flagging of responses outside of expected ranges. Adaptive and forced protocols are also implemented into CRFs. For example, anthropometric measurements for the first replicate need to be entered before the next replicate fields will be displayed, meaning a full replicate of the measurement protocol is followed instead of basic remeasurements with participants and equipment in the same position. Adaptive protocols and instructions are also included. For example, for certain anthropometric measurements such as triceps skinfold, the research assistant determines the participant’s dominant arm, triggering ConnEDCt to prompt the research assistant to use the participant’s other, nondominant arm. Furthermore, warning pop-ups (logic checks) for impossible or nonbiologically plausible data (such as body weight entered or calculated as a negative number) were incorporated into ConnEDCt, allowing real-time data correction during data collection, minimizing challenges in ascertaining errors during data cleaning and analysis. Queries, raised by research assistants and/or data managers concurrently with the study or after data collection is completed, can be settled by data managers who have form modification privileges. ConnEDCt includes an audit trail to monitor when data were modified and by whom.

### Conclusions

ConnEDCt, an EDC system, is an ideal tool for research studies, particularly those with complex protocols in settings where internet access is limited. In addition to mitigating the time required and error-prone nature of paper-based data collection methods, ConnEDCt represents a fixed framework that is adaptable to a variety of study designs. As demonstrated in a variety of settings, the ConnEDCt EDC system has been integral to carrying out several studies, all diverse in design and setting, types of participants, and overall goals for the research. Compared with other EDC tools [1,2,13-24], ConnEDCt’s benefits, including its utility as a mobile system, the ability to collect data without internet access, customization options for specific study designs, and data security, are comparable with systems such as REDCap and other systems [3,25,26], while at the same time serving complex protocols more precisely than other systems. An EDC system can be made as a straightforward framework that is adaptable for the successful management and completion of almost all kinds of field-based research studies and allows for easy export and transfer of collected data into statistical processing software for further analyses.

## Abbreviations

CFR: Code of Federal Regulations
CRF: case report form
EDC: electronic data capture
FDA: Food and Drug Administration
MBFC: multiple biofortified food crops
QFS: quadruple fortified salt
RCT: randomized controlled trial
REDCap: Research Electronic Data Capture
